# Author Correction: Identification of epilepsy-associated neuronal subtypes and gene expression underlying epileptogenesis

**DOI:** 10.1038/s41467-020-19869-5

**Published:** 2020-11-19

**Authors:** Ulrich Pfisterer, Viktor Petukhov, Samuel Demharter, Johanna Meichsner, Jonatan J. Thompson, Mykhailo Y. Batiuk, Andrea Asenjo-Martinez, Navneet A. Vasistha, Ashish Thakur, Jens Mikkelsen, Istvan Adorjan, Lars H. Pinborg, Tune H. Pers, Jakob von Engelhardt, Peter V. Kharchenko, Konstantin Khodosevich

**Affiliations:** 1 Biotech Research and Innovation Centre (BRIC), Faculty of Health and Medical Sciences, University of Copenhagen, 2200 Copenhagen, Denmark; 2 Department of Biomedical Informatics, Harvard Medical School, Boston, MA 02115 USA; 3 Institute of Pathophysiology, University Medical Center of the Johannes Gutenberg University Mainz, Mainz, Germany; 4 Novo Nordisk Foundation Center for Basic Metabolic Research, Faculty of Health and Medical Sciences, University of Copenhagen, 2200 Copenhagen, Denmark; 5 Department of Neurology and Neurobiology Research Unit, Copenhagen University Hospital, Rigshospitalet, Copenhagen, Denmark; 6 Department of Anatomy, Histology and Embryology, Semmelweis University, Budapest, Hungary; 7 Neurobiology Research Unit, Copenhagen University Hospital, Rigshospitalet, 2200 Copenhagen, Denmark; 8 Epilepsy Clinic, Department of Neurology, Copenhagen University Hospital, Rigshospitalet, 2200 Copenhagen, Denmark

**Keywords:** RNA sequencing, Epilepsy, Molecular neuroscience

Correction to: *Nature Communications* 10.1038/s41467-020-18752-7, published online 7 October 2020.

The original version of this Article contained an error in the spelling of the author Andrea Asenjo-Martinez, which was incorrectly given as Andrea Asenjo Martinez.

This has now been corrected in both the PDF and HTML versions of the Article.

