# Supplementary material for: Identification of epilepsy-associated neuronal subtypes and gene expression underlying epileptogenesis
Source: Nat Commun. 2020 Oct 7;11:5038. doi: 10.1038/s41467-020-18752-7 (PMC7541486; doi:10.1038/s41467-020-18752-7)
Supplement: Supplementary file 1 — Supplementary Information [file 41467_2020_18752_MOESM1_ESM.pdf]

Supplementary Fig. 1

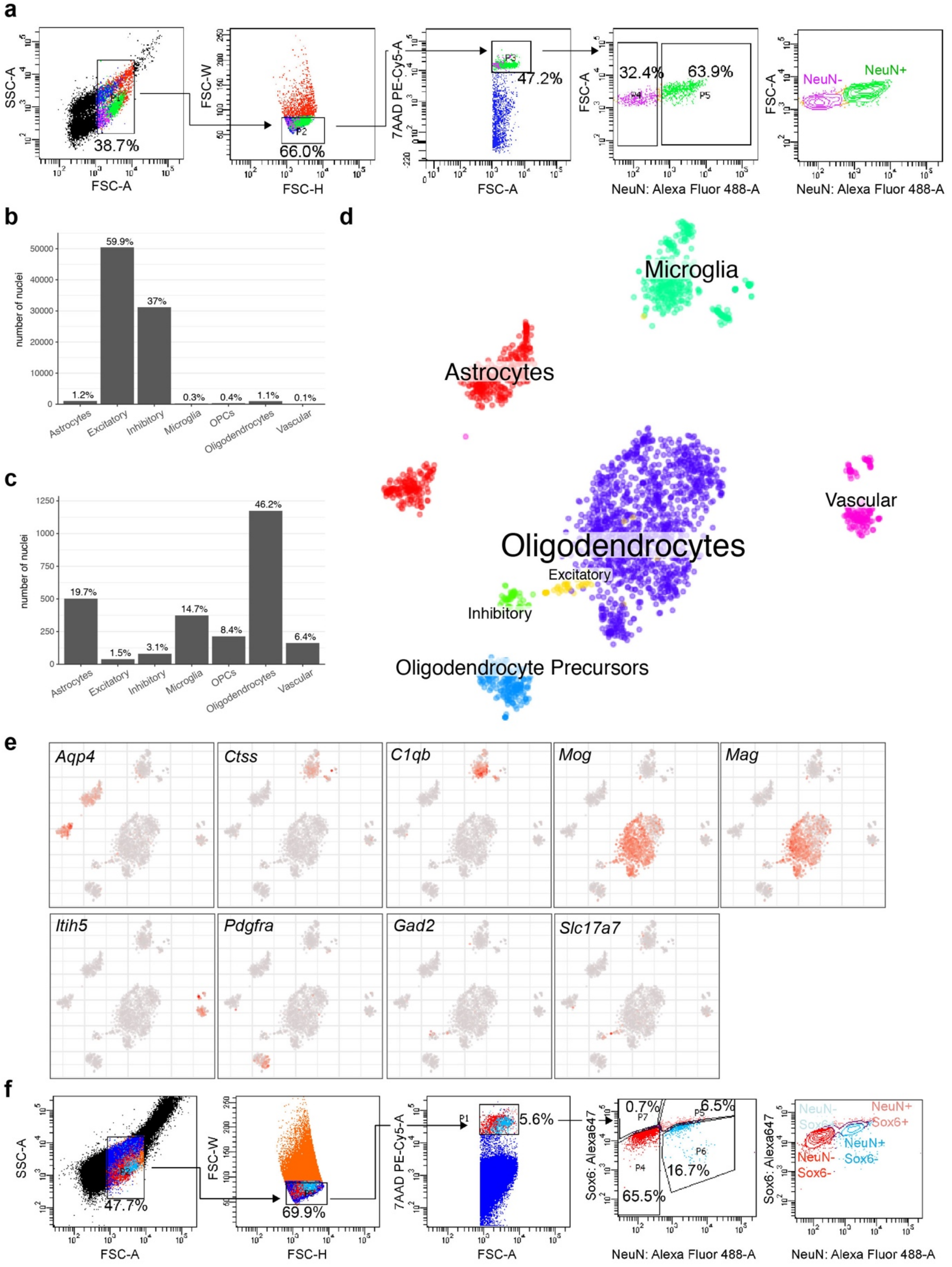

Supplementary Figure 1. Validation of isolation strategy of neuronal and non-neuronal nuclei from human temporal cortex. a. Representative FANS plots for flow cytometric isolation of NeuN+ neuronal nuclei and NeuN- non-neuronal fraction for sample CNT04.

Arrows indicate sequential gating strategy using dot plots with summarizing contour plot illustrating NeuN+ and NeuN- fractions. Numbers in percent indicate population size relative to the respective parent gate. Each sample was processed separately for FANS and 10X Genomics cDNA library preparation. b, c. Number of nuclei with neuronal or non-neuronal identity isolated from the NeuN+ (b) or NeuN- (c) fraction, respectively. Percentage of the total is shown on the top of the bars. d. UMAP representation and cell-type annotation demonstrate that the vast majority of nuclei in NeuN- fraction belong to non-neuronal cell-types. e. Visualization of the log-normalized expression of the marker genes for the main non-neuronal and neuronal cell-types reveals only residual presence of neuronal nuclei in the NeuN- fraction. f. Representative plots for flow cytometric isolation of NeuN+/Sox6+ and NeuN+/Sox6- neuronal nuclei for sample TLE1 for Smart-seq2 using NeuN and Sox6 double staining.

Supplementary Fig. 2

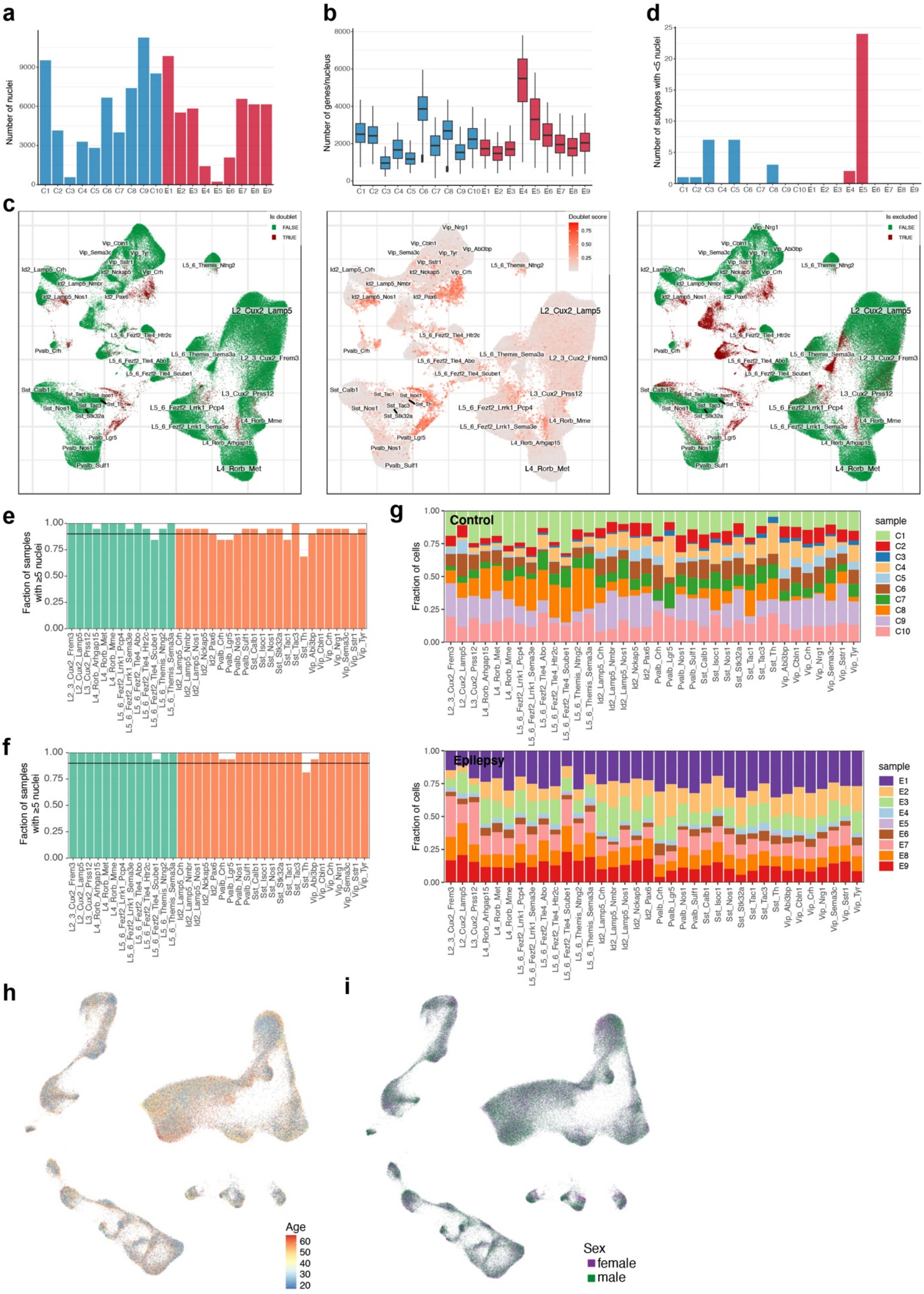

Supplementary Figure 2. Quality control (QC) of RNA sequencing libraries and per-sample analysis of subtype representation. a. Number of nuclei passing QC and used for

downstream analysis per brain sample. b. Distribution of the number of genes expressed per nucleus per brain sample shown with boxplots. c. Removal of doublets was step-wise – first by Scrublet (in left panel red dots are doublets, middle panel shows doublet score) and then by using cell type specific markers to identify mixed clusters (right panel, red dots indicate all excluded nuclei) d. Quantification of subtypes with <5 nuclei for each brain sample. Plots (a) and (d) show that number of cells in the samples C3 and E5 is too low, and that C5 misses some cell types. Thus, C3, C5 and E5 were excluded from further analysis. e, f. Fraction of samples with at least 5 nuclei shown for each subtype, before (e) and after (f) removal of C3, C5 and E5 samples. Black horizontal line represents 90% of samples. g. Fraction of cells assigned to annotated neuronal subtypes visualized by brain sample demonstrated representation of all subtypes in the vast majority of brain samples. h, i. UMAP embedding of nuclei colored by age (h) and sex (i) of the individual samples showed a minor impact of both parameters on the transcriptional identity of annotated neuronal subtypes.

Supplementary Fig. 3

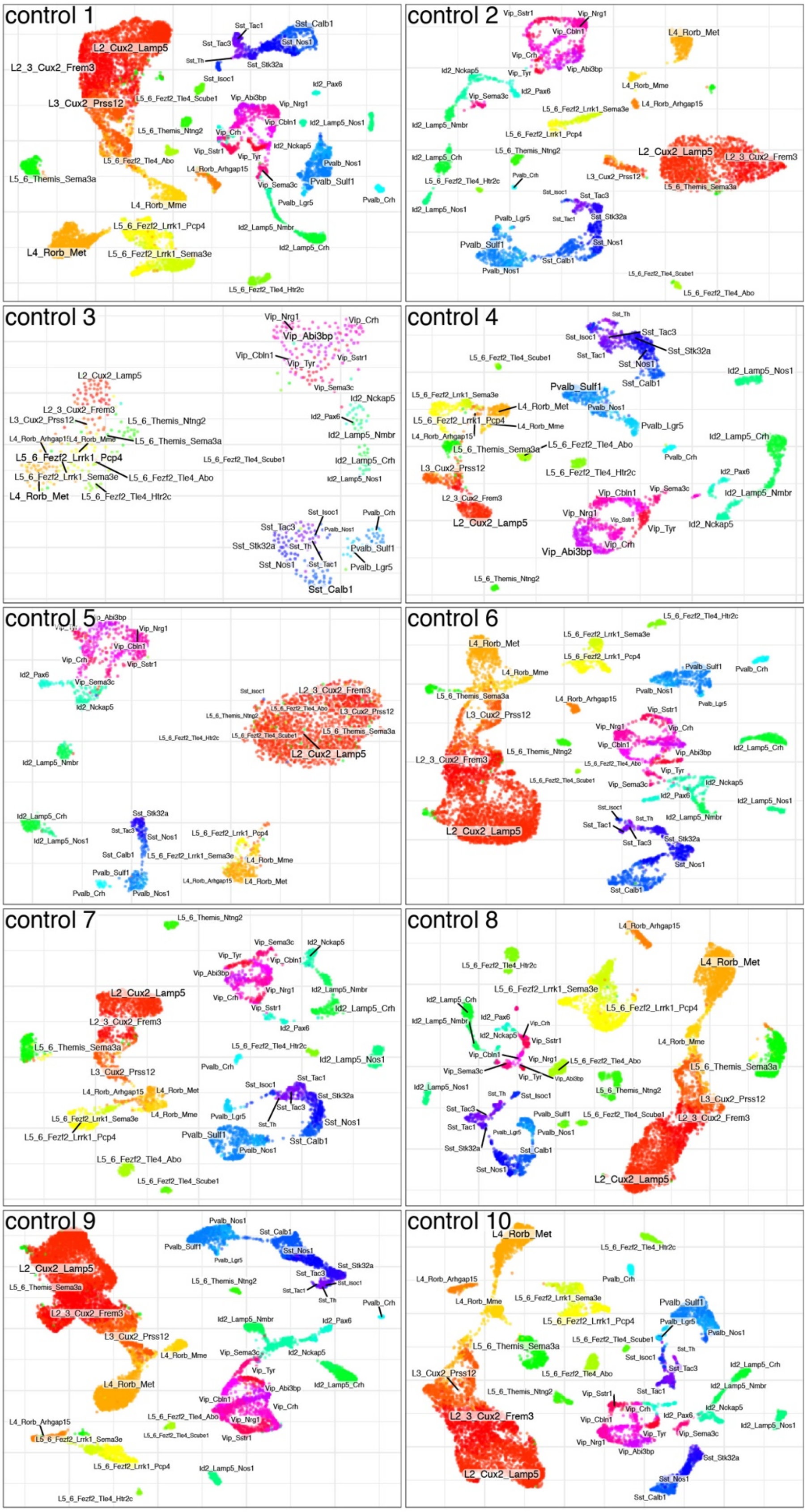

Supplementary Figure 3. UMAP embedding and cell type annotation visualized for individual control samples.

Supplementary Fig. 4

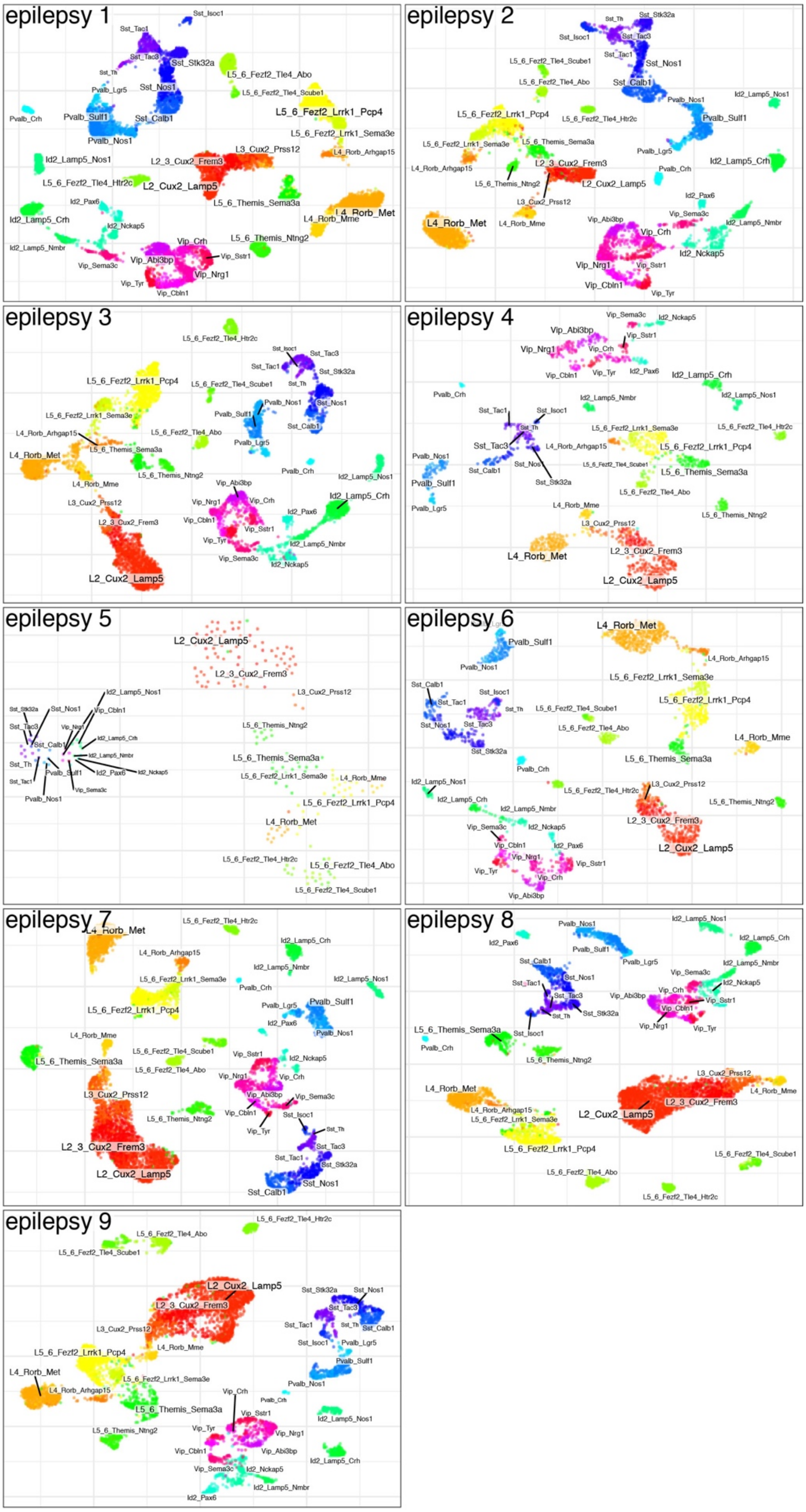

Supplementary Figure 4. UMAP embedding and cell type annotation visualized for individual epilepsy samples.

Supplementary Fig. 5

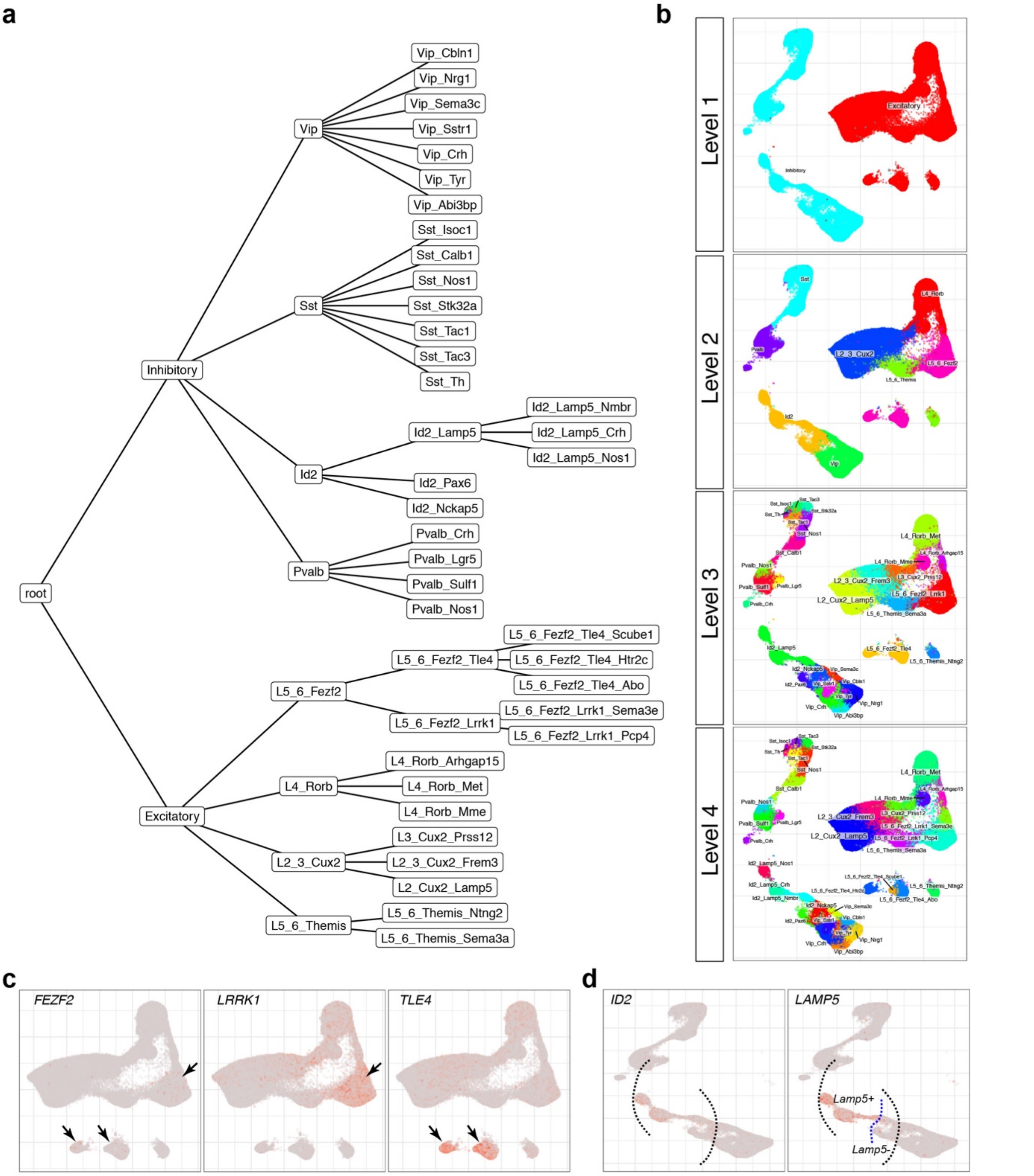

Supplementary Figure 5. Hierarchical annotation for neuronal nuclei from epileptic and non-epileptic temporal cortex. a. Hierarchical tree showing four levels of annotation. b. UMAP plots visualizing four levels of annotation. c. Markers allowing to subdivide L5-6_Fezf2 family of principal neurons into L5-6_Fezf2_Lrrk1 and L5-6_Fezf2_Tle4 subfamilies. d. Markers allowing to subdivide Id2 family of GABAergic interneurons into Id2_Lamp5-positive and Id2_Lamp5-negative subfamilies.

Supplementary Fig. 6

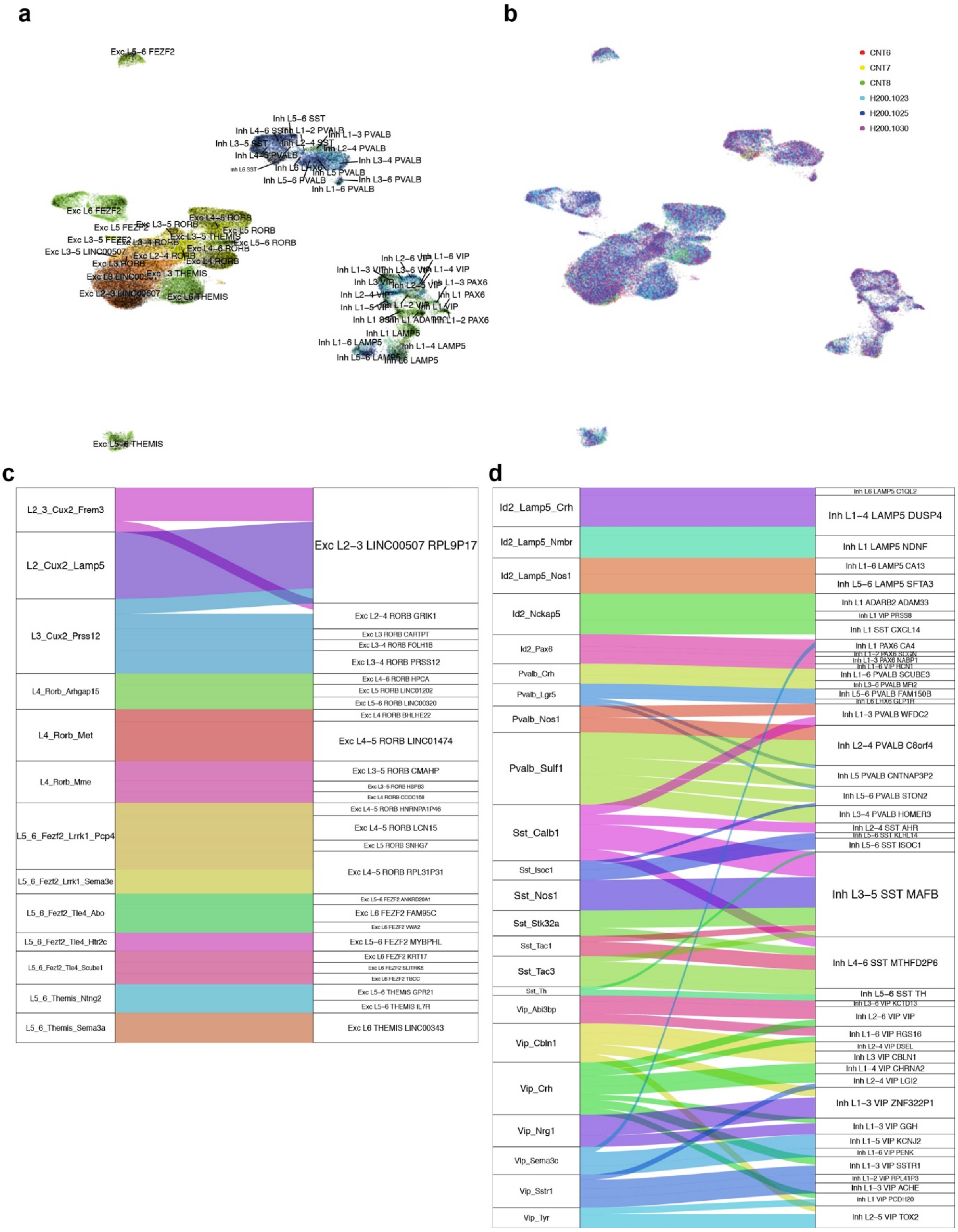

Supplementary Figure 6. Neuronal annotation in this study matches previously reported annotation of healthy human temporal cortex. a. Conos alignment of our and Hodge et al.[7]

datasets (sequenced by 10X Genomics and Smart-seq2 methods, respectively), colored by annotated subtype, where black dots label neuronal nuclei from Hodge et al. dataset. b. Alignment of our and Hodge et al. datasets, colored by sample. c, d. Matching of our and Hodge et al. annotations demonstrates good correspondence between the transcriptomic subtypes in two studies. The thickness of the colored lines that link our and Hodge et al. annotations is proportional to the squared root of the number of cells in the matched segment, and the coloring of the lines corresponds to subtypes from our annotation (the left columns). Higher number of subtypes in Hodge et al. study [7] might be explained by higher resolution in transcriptome that is provided by Smart-seq2 method for single nucleus RNA sequencing or by iterative clustering method that was utilized in Hodge et al. [7] to define transcriptomic subtypes.

Supplementary Fig. 7

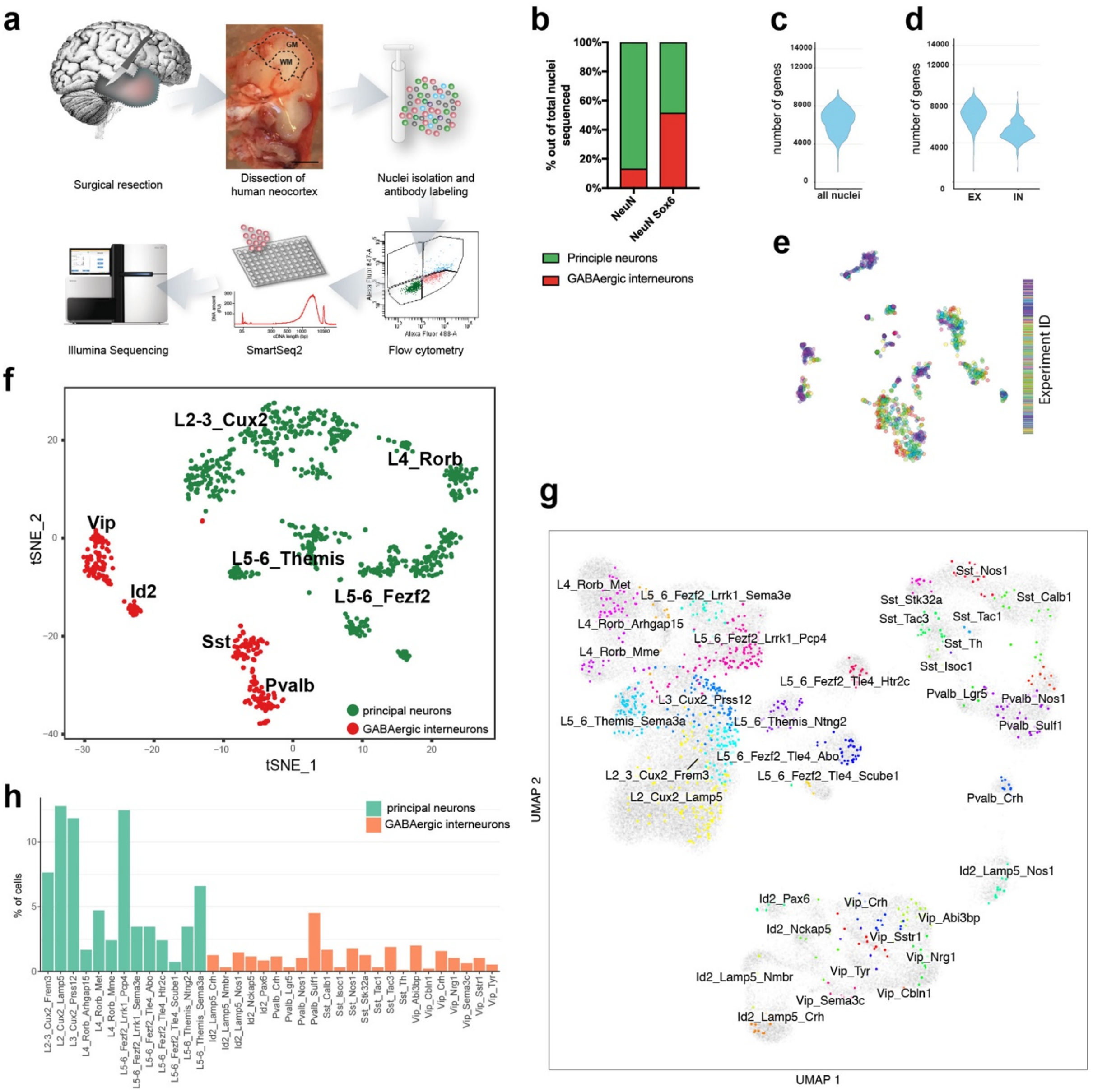

Supplementary Figure 7. Neuronal type annotations and neuronal type representation is independent of single nucleus transcriptomics method. a. Schematic representation of the experimental outline to perform Smart-seq2-based single nucleus RNA-sequencing on FANS-isolated nuclei. b. Single nuclei were isolated based on NeuN (all neurons) and NeuN/Sox6 (enrichment for GABAergic interneurons) labelling. NeuN/Sox6 double labelling increased the fraction of GABAergic interneurons by 4-fold compared to NeuN single labelling. c, d. Number of detected genes per nucleus, total (c) and separated (d) into principal neurons (EX) and GABAergic interneurons (IN). e. t-SNE distribution of 1,114 single nuclei sequenced by Smart-seq2 shows good overall distribution of nuclei regardless of the day they were processed (experiment ID). Enrichment for NeuN/Sox6-double positive nuclei for the last set of

experiments increased the proportion of GABAergic interneuron nuclei (blue-purple colors). No batch effects were observed. f. t-SNE representation of neuronal nuclei separated into principal neurons (red) and GABAergic interneurons (green). g. Joint embedding of Smart-seq2 processed nuclei with 10X dataset using previously established annotations shows good alignment of neuronal nuclei processed by two methods. Colored points correspond to Smart-seq2 cells with colors representing cell type annotation; 10X cells are colored in grey. h. Quantification of subtype identity of Smart-seq2 processed nuclei reveals that all previously identified neuronal type signatures are present and confirm that neuronal type annotations are independent of the method used.

Supplementary Fig. 8

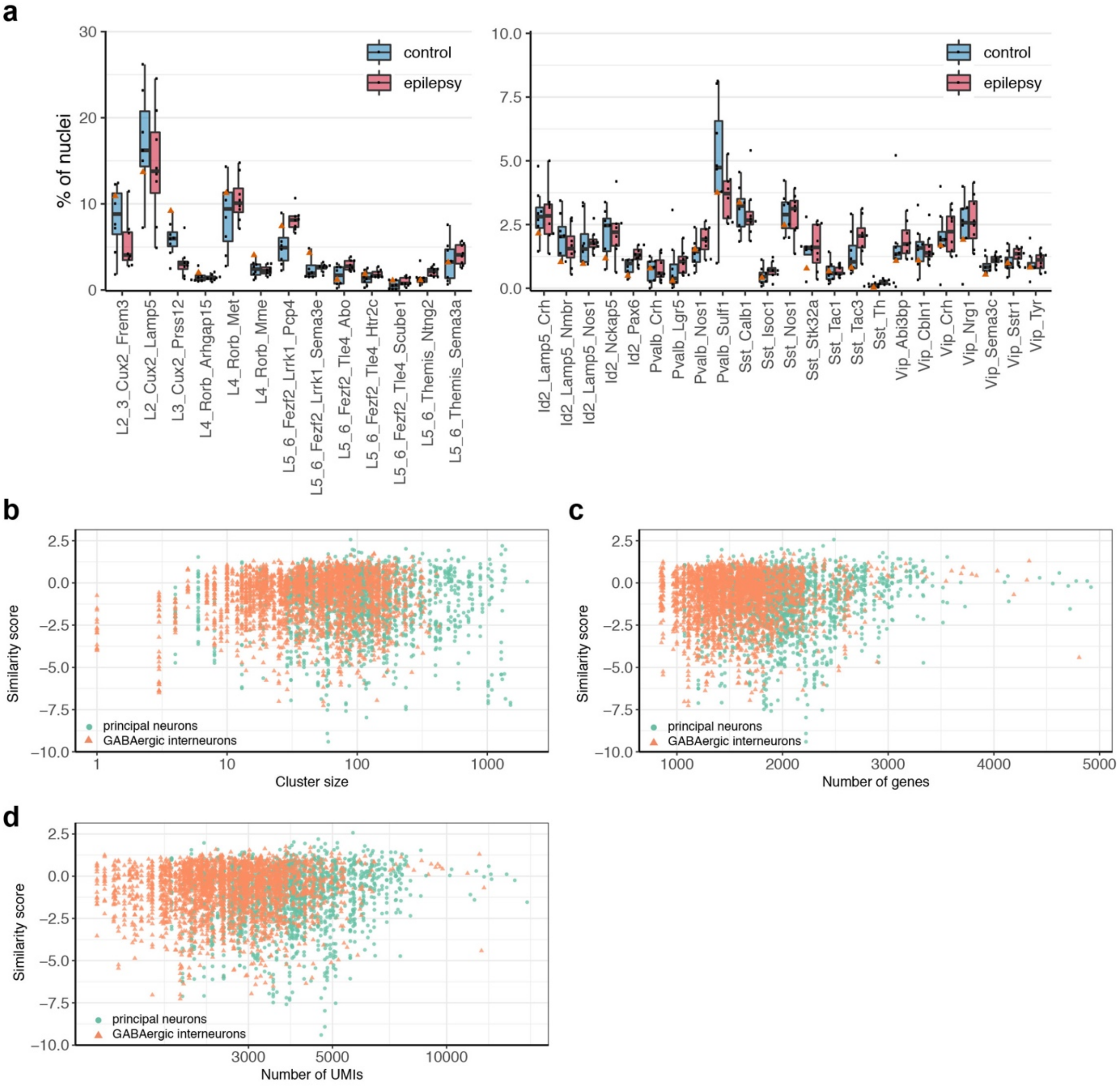

Supplementary Figure 8. Proportion of neuronal subtype recovery and gene expression similarity score analysis. a. Percentage of nuclei per subtype of principal neurons and GABAergic interneurons reveals that the recovery of neuronal subtypes was highly similar between epileptic and non-epileptic samples. As quality control, non-epileptic samples contained a biopsy (labeled by orange triangle), and distribution of proportions for neuronal subtypes in the biopsy matched well with the rest of non-epileptic samples that were obtained by autopsy. b, c, d. Gene expression similarity score does not depend on cluster size, number of genes and number of UMIs per subtype, respectively.

Supplementary Fig. 9

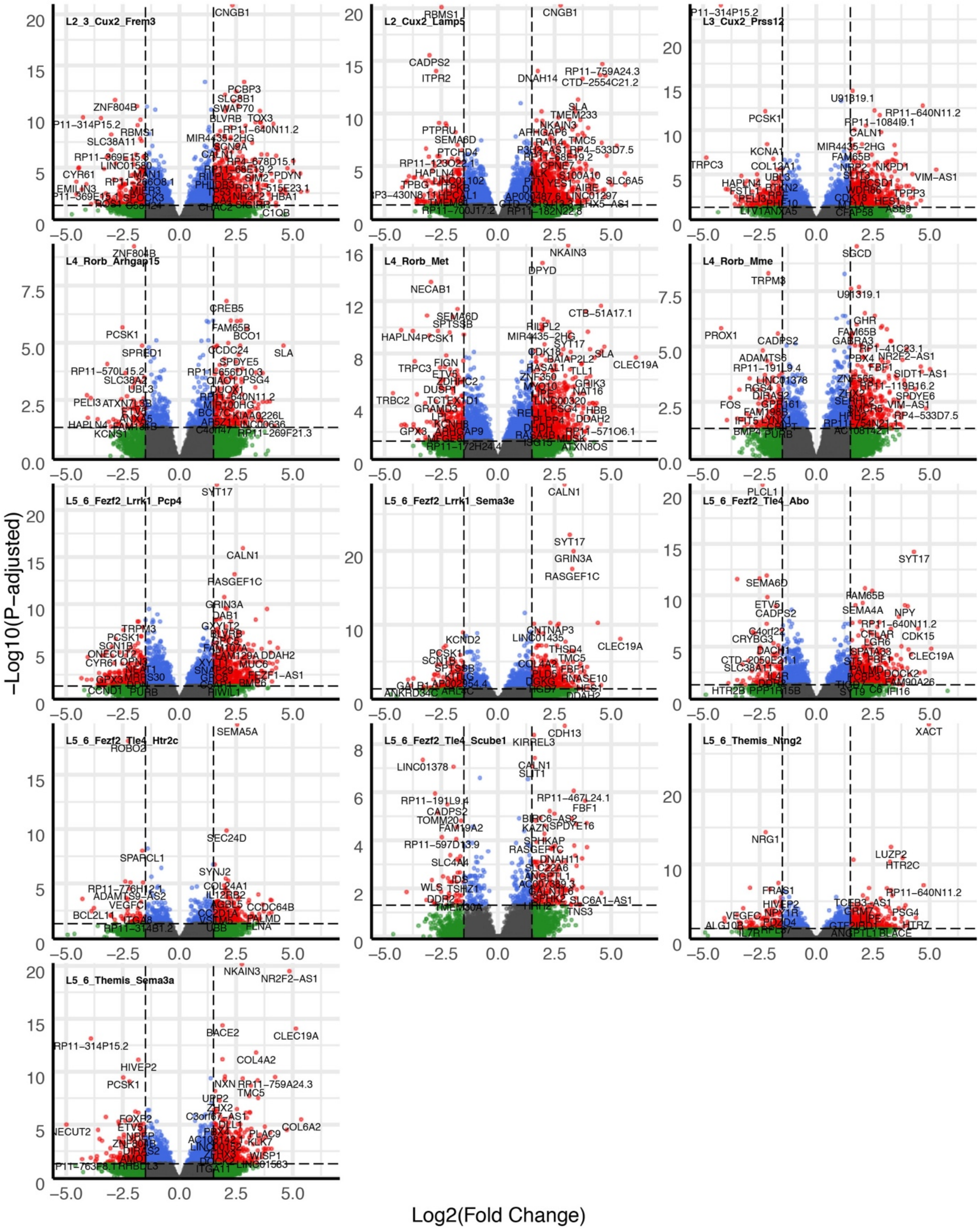

Supplementary Figure 9. Volcano plots for control vs epilepsy DE genes in each subtype of principal neurons. On X and Y axes, Log2 fold change of gene expression and -Log10(p-adjusted) for significance of expression changes, respectively. Horizontal dashed line shows -Log10(p-adjusted) = 1.25, two vertical dashed lines show Log2 fold change = 1.25/-1.25. Colors indicate, green: -Log10(p-adjusted) < 1.25, Log2 fold change >2.5 or <-2.5; red: -

Log10(p-adjusted) > 1.25, Log2 fold change >2.5 or <-2.5; blue: -Log10(p-adjusted) > 1.25, Log2 fold change <2.5 and >-2.5; grey: -Log10(p-adjusted) > 1.25, Log2 fold change <2.5 and >-2.5. Names for some of the genes that have top -Log10(p-adjusted) values and highest/lowest Log2 fold change values are shown on the plots.

Supplementary Fig. 10

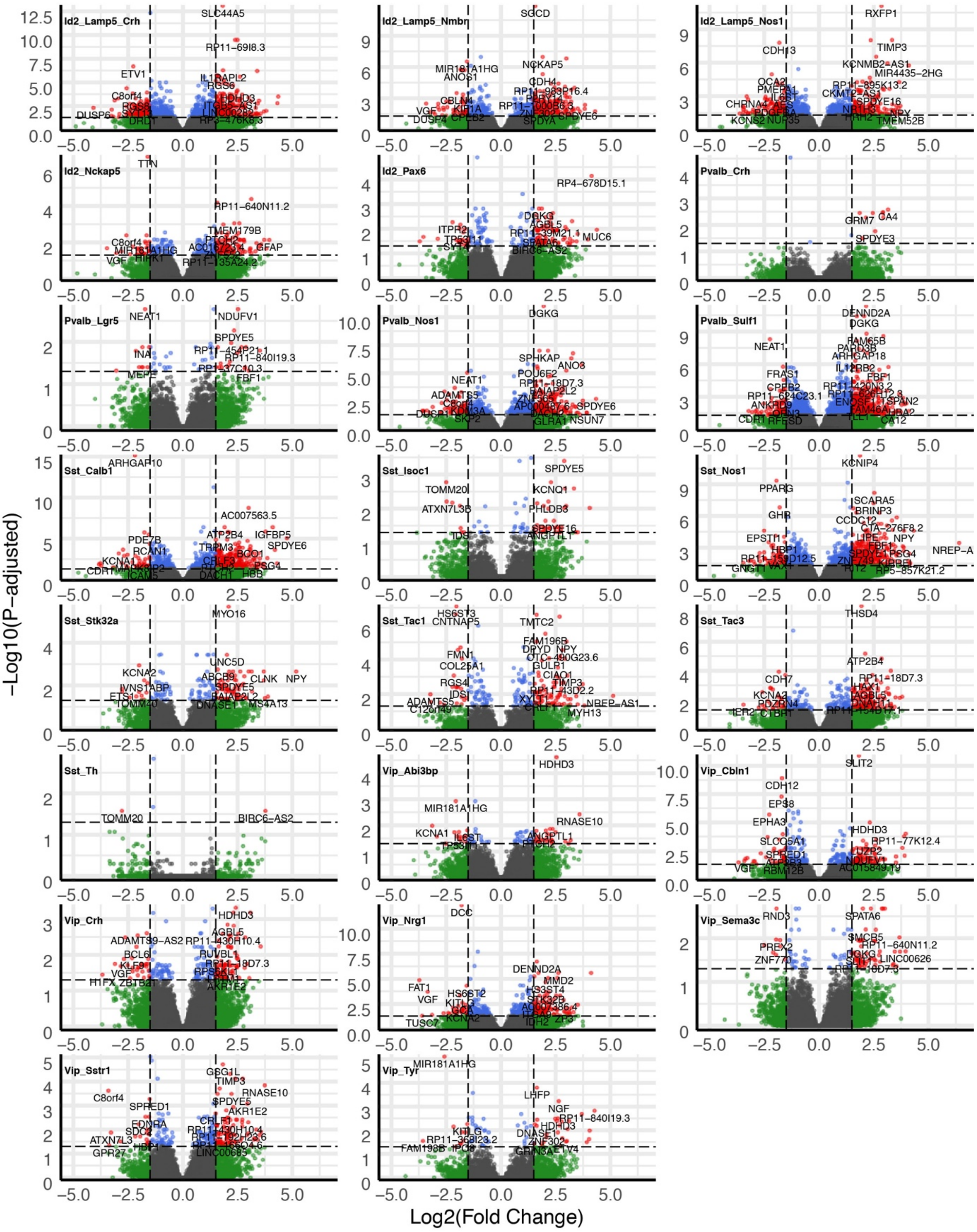

Supplementary Figure 10. Volcano plots for control vs epilepsy DE genes in each subtype of GABAergic interneurons. On X and Y axes, Log2 fold change of gene expression and -Log10(p-adjusted) for significance of expression changes, respectively. Horizontal dashed line shows -Log10(p-adjusted) = 1.25, two vertical dashed lines show Log2 fold change = 1.25/-1.25. Colors indicate, green: -Log10(p-adjusted) < 1.25, Log2 fold change >2.5 or <-2.5; red:

-Log10(p-adjusted) > 1.25, Log2 fold change >2.5 or <-2.5; blue: -Log10(p-adjusted) > 1.25, Log2 fold change <2.5 and >-2.5; grey: -Log10(p-adjusted) > 1.25, Log2 fold change <2.5 and >-2.5. Names for some of the genes that have top -Log10(p-adjusted) values and highest/lowest Log2 fold change values are shown on the plots.

Supplementary Fig. 11

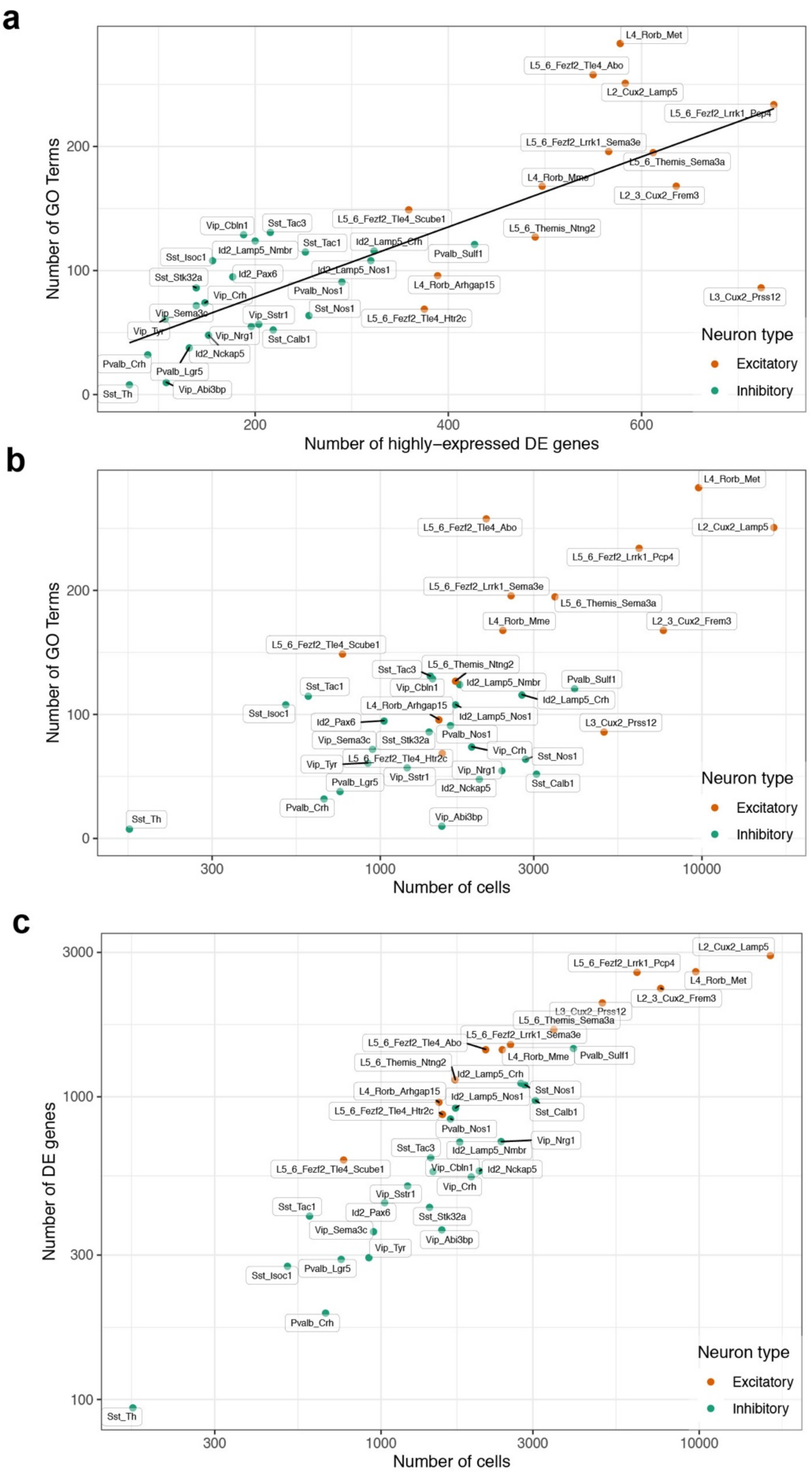

Supplementary Figure 11. Correlation of different parameters for GO term analysis. a. Correlation plot between number of GO terms and number of highly-expressed DE genes. b.

Correlation for number of GO terms and number of nuclei sequenced for a subtype. c. Correlation between number of highly-expressed DE genes and number of nuclei sequenced for a subtype. Line in (a) was obtained with robust linear regression of the corresponding variables.

Supplementary Fig. 12

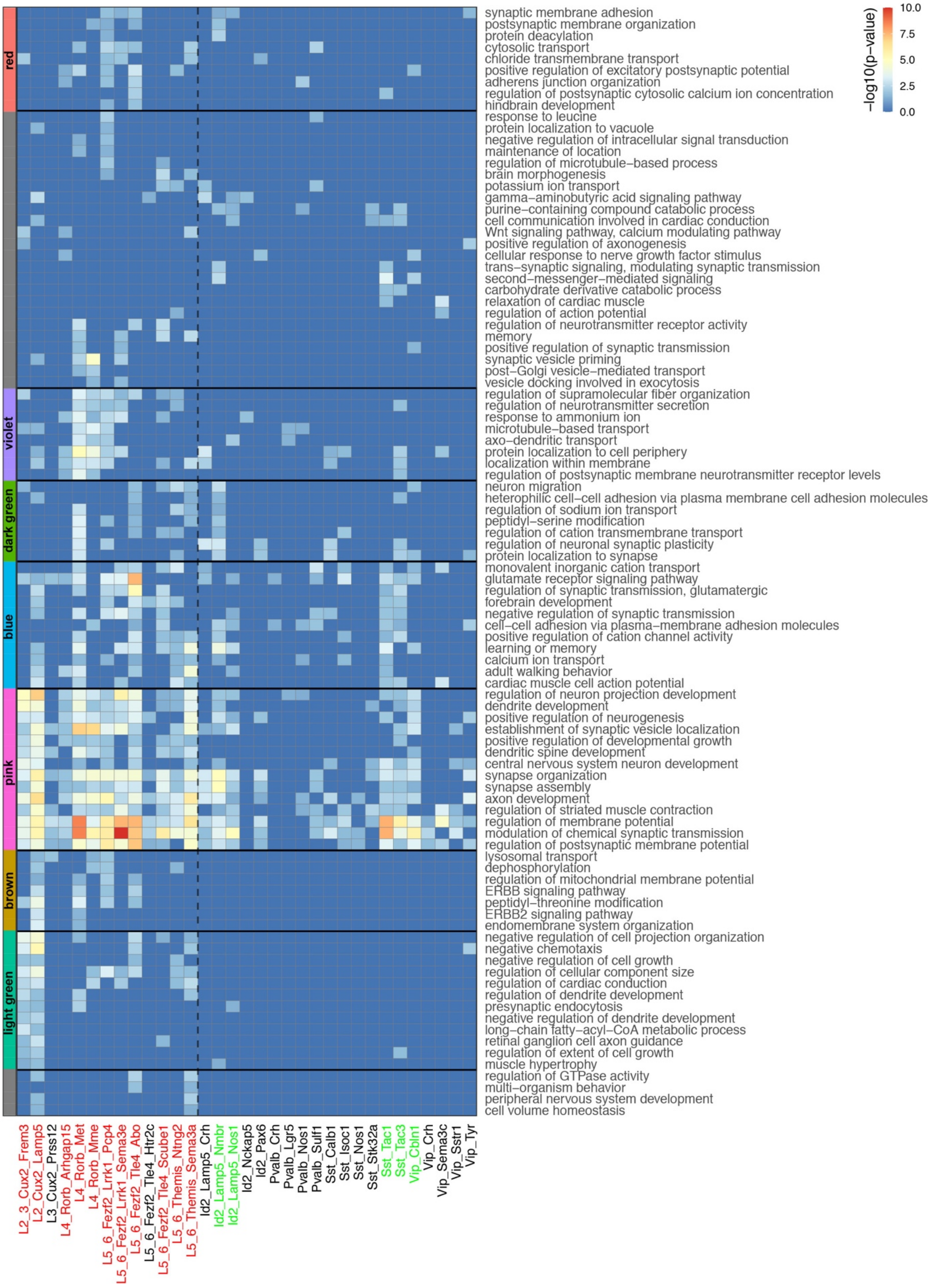

Supplementary Figure 12. GO Term clustering by level of enrichment. Clustering shows similar enrichment patterns for specific GO terms in distinct neuronal subtypes indicative of

common transcriptomic shifts underlying epilepsy with the most significant alterations being involved in neural circuit re-organization and neurotransmission (Pink cluster). Rows correspond to GO terms, ordered according to hierarchical clustering. Columns correspond to cell types. Highlighted in red or green are principal neuron or GABAergic interneuron subtypes, which have the largest enrichment in clustered GO terms. Heatmap colors represent -log10 of adjusted *p*-values of the overrepresentation test, trimmed with upper boundary of 10. The largest clusters are highlighted with bold black lines and colors on the left vertical color bar.

Supplementary Fig. 13

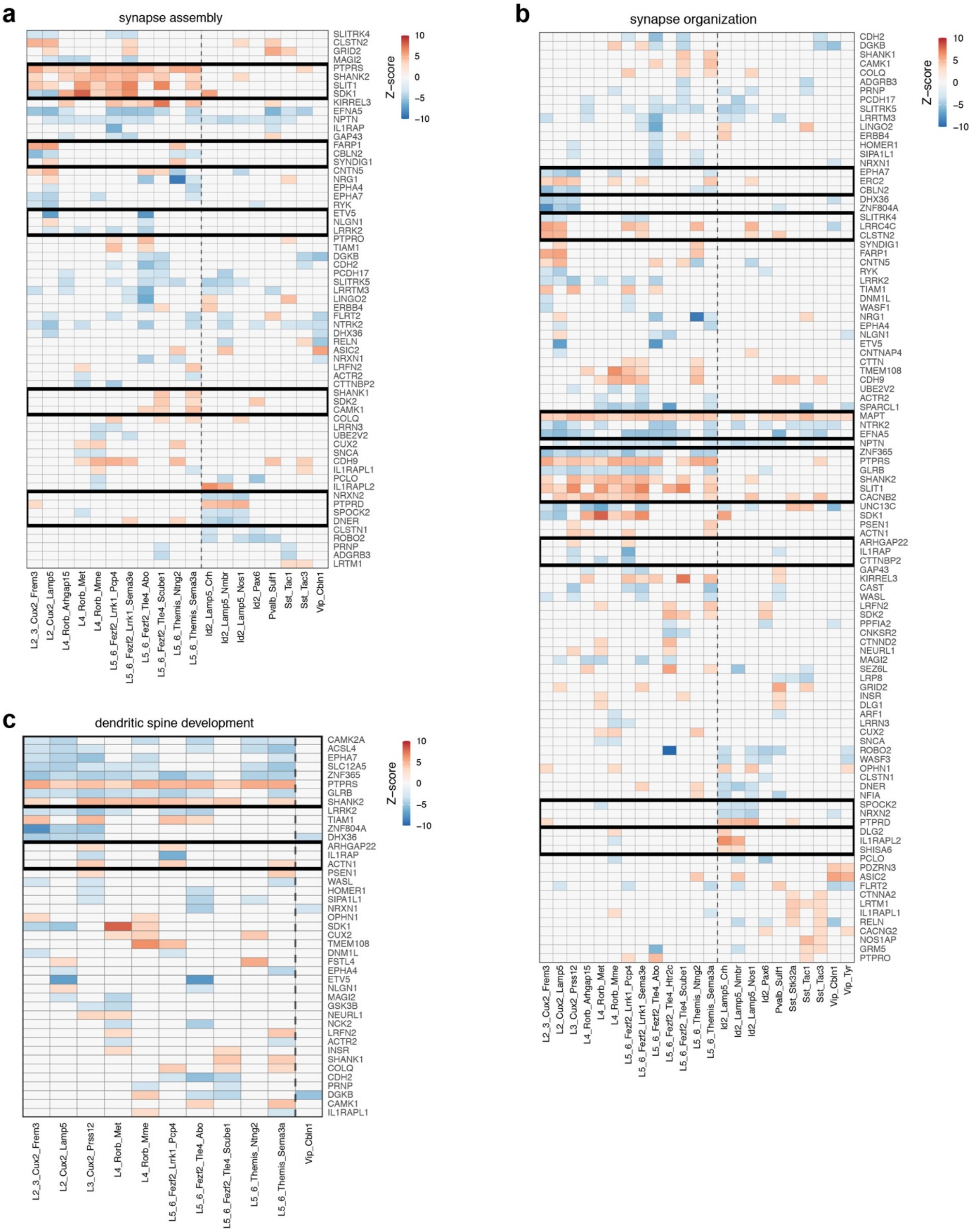

Supplementary Figure 13. Neuronal subtype clustering based on similarity of DE gene expression within the GO terms of the Blue cluster, part 1. a, b, c. Heatmaps of DE genes for GO terms *synapse assembly, synapse organization*, and *dendritic spine development*. Rows correspond to genes of the specific GO term shown on the top of the heatmap that were enriched in at least one of the annotated subtypes. Rows are ordered based on L_1-distance of the z-score

patterns. Columns correspond to neuronal subtypes, for which at least one of the genes was enriched. Colors represent z-scores of the differential expression between control and epilepsy datasets. All z-scores with absolute values < 3 were set to 0, as such genes were excluded from the enrichment analysis. Bold black rectangles highlight clusters of genes, selected based on L_1-distance of their z-scores.

Supplementary Fig. 14

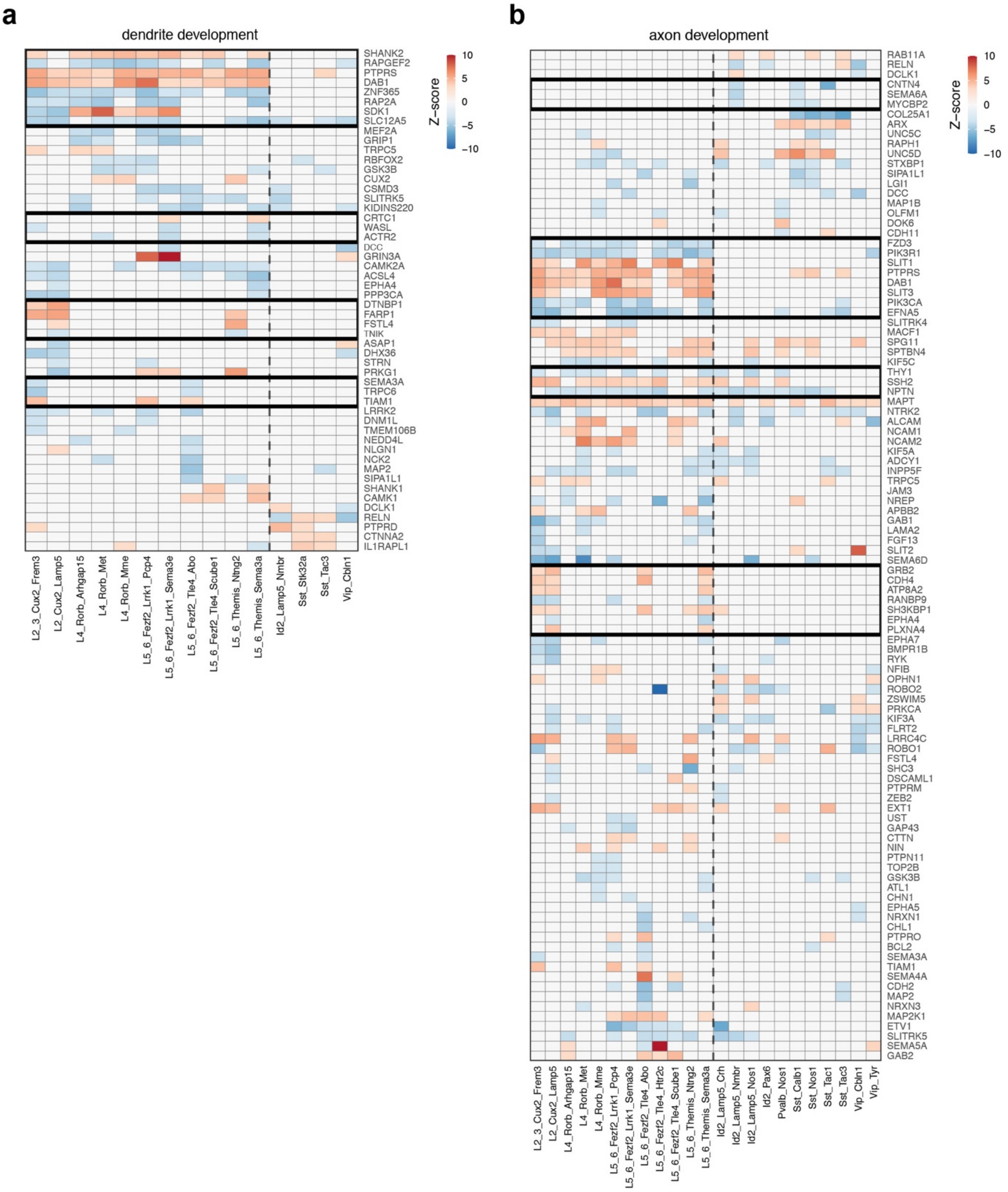

Supplementary Figure 14. Neuronal subtype clustering based on similarity of DE gene expression within the GO terms of the Blue cluster, part 2. a, b. Clustering of DE genes for GO terms *dendrite development*, *axon development*. The formatting is the same as in Supplementary Fig. 13.

Supplementary Fig. 15

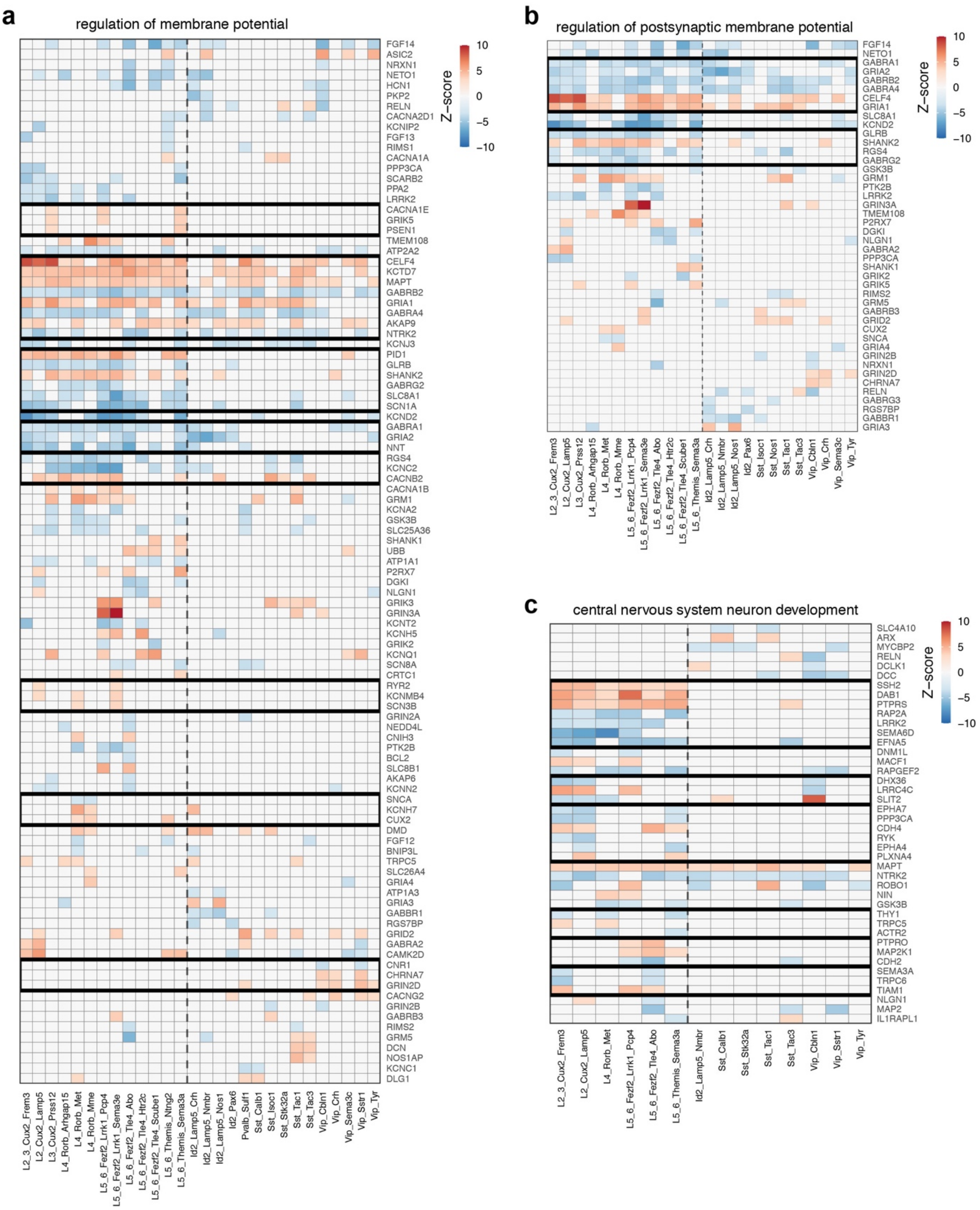

Supplementary Figure 15. Neuronal subtype clustering based on similarity of DE gene expression within the GO terms of the Blue cluster, part 3. a, b, c. Clustering of DE genes for GO terms *regulation of membrane potential*, *regulation of postsynaptic membrane potential* and *central nervous system neuron development*. The formatting is the same as in Supplementary Fig. 13.

Supplementary Fig. 16

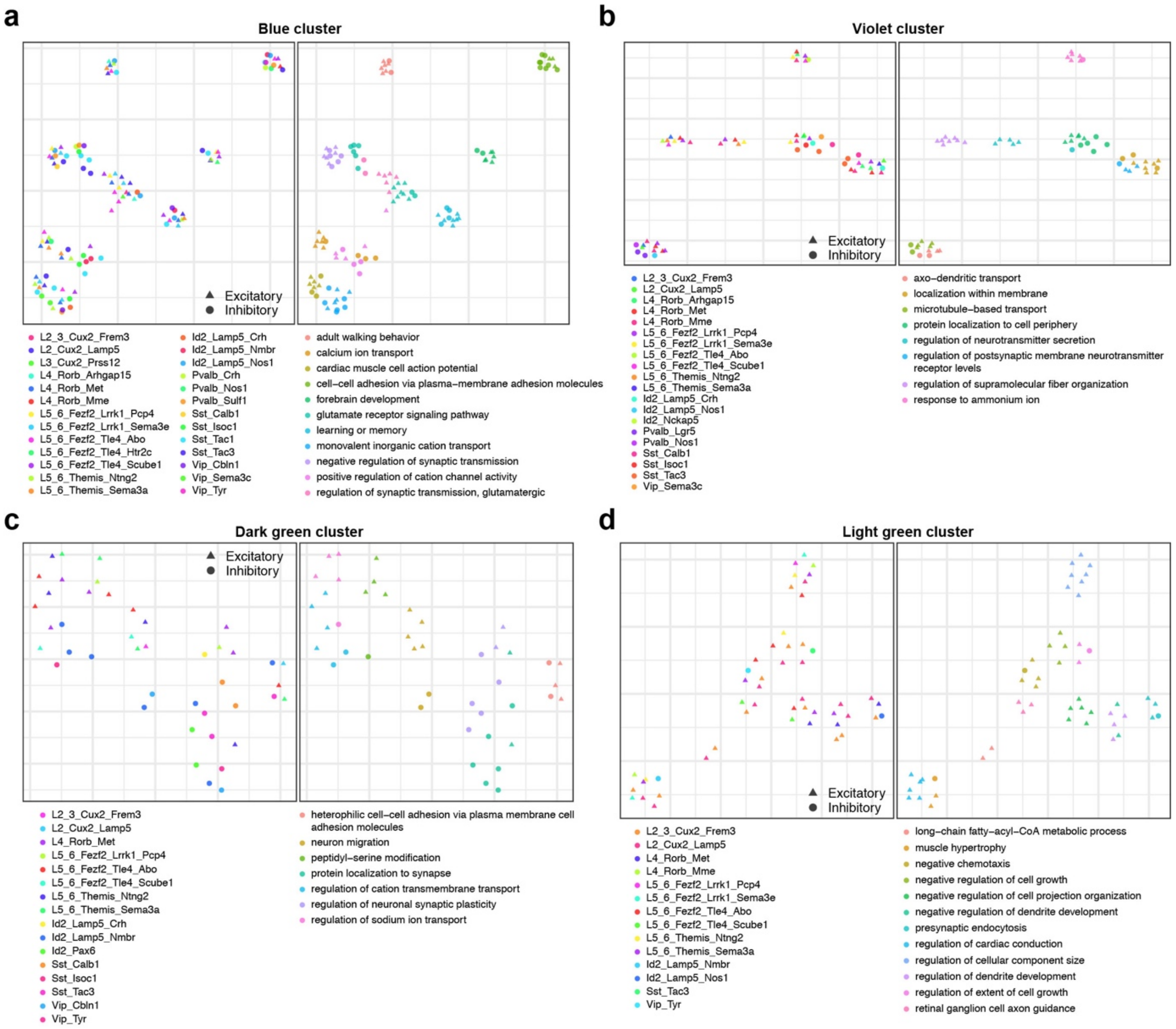

Supplementary Figure 16. GO-term clustering by their level of enrichment per subtype for the Blue, Violet, Dark green and Light green clusters. a, b, c, d. GO-term clustering for the Blue, Violet, Dark green and Light green clusters, respectively. For more detailed explanation of clustering procedure see the legend for Fig. 3c and the methods.

Supplementary Fig. 17

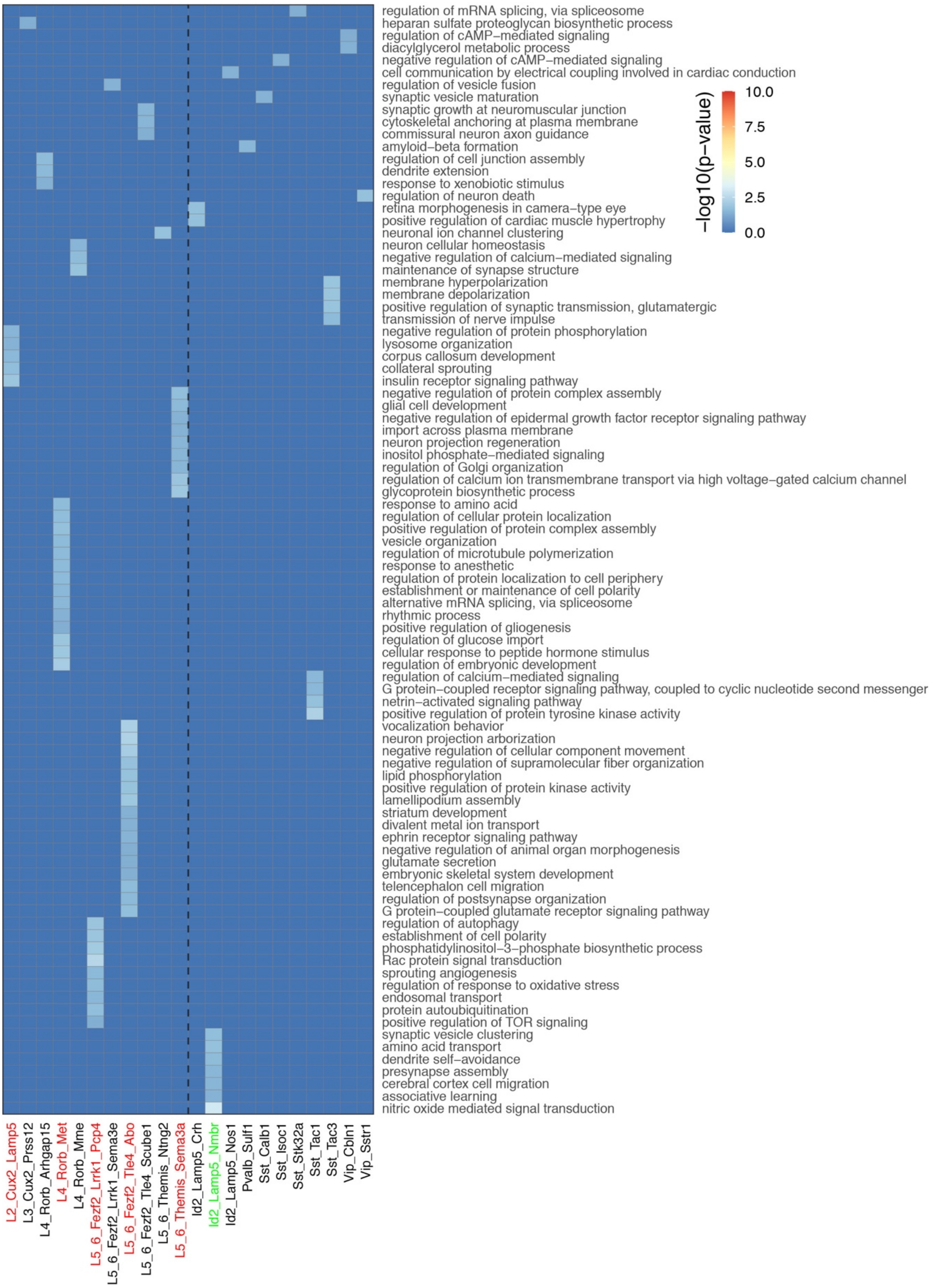

Supplementary Figure 17. Unique GO terms that are enriched in specific subtypes of principal neurons and GABAergic interneurons. The formatting is the same as in Supplementary Fig. 12.

Supplementary Fig. 18

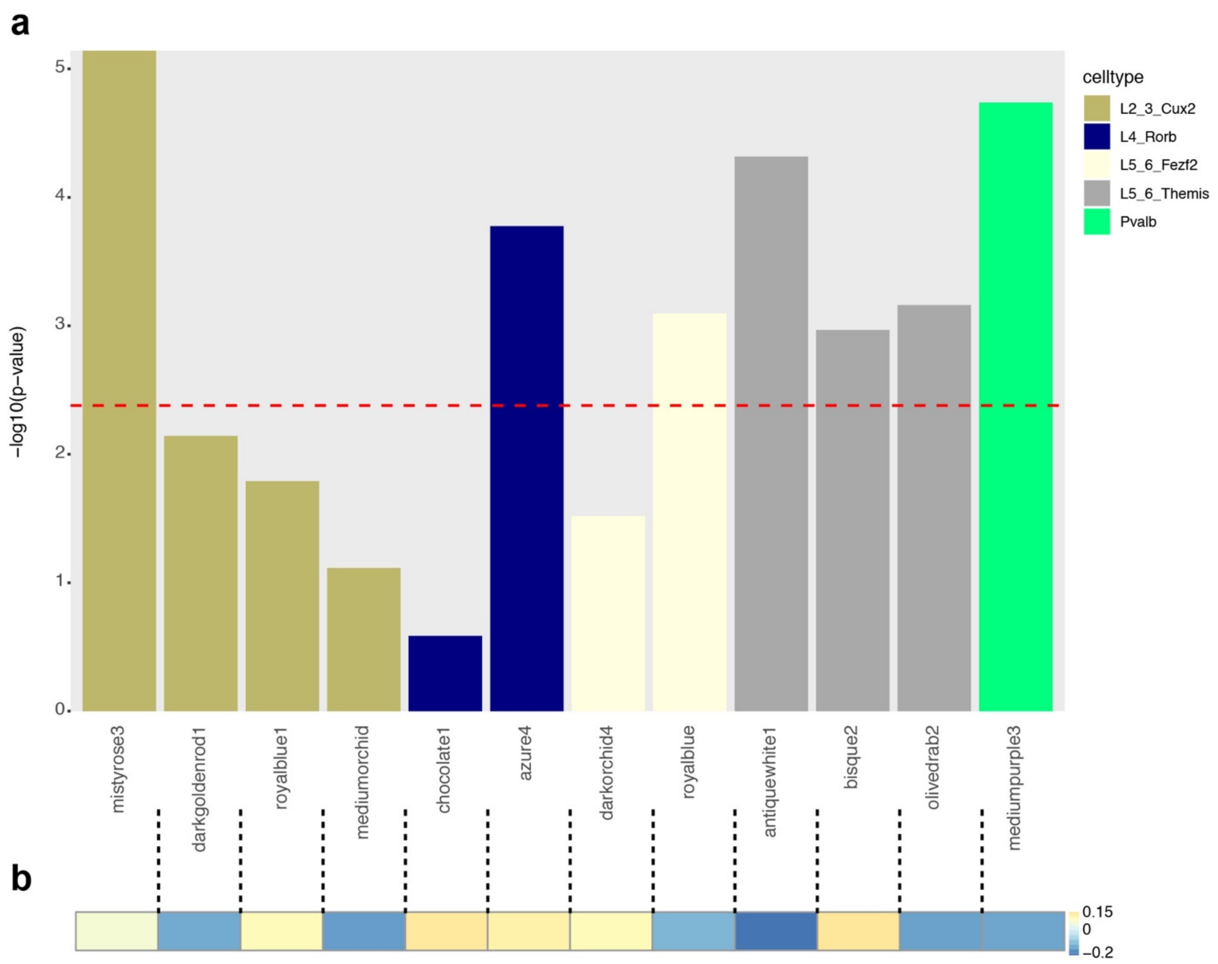

Supplementary Figure 18. rWGCNA gene module association with epilepsy. a. Co-expression enrichment of 12 prioritized gene modules with curated epilepsy-associated gene list (Supplementary Table 7, Methods), grouped by the level 2 cell type in which the module was detected. b. Linear model coefficients for epilepsy with module expression as outcome. The units are the sum of normalized module gene expression weighted by rWGCNA kIM scores normalized to sum to 1.
