# Supplementary material for: Identification of epilepsy-associated neuronal subtypes and gene expression underlying epileptogenesis
Source: Nat Commun. 2020 Oct 7;11:5038. doi: 10.1038/s41467-020-18752-7 (PMC7541486; doi:10.1038/s41467-020-18752-7)
Supplement: Supplementary file 3 — Reporting Summary [file 41467_2020_18752_MOESM3_ESM.pdf]

nature research

Corresponding author(s): Konstantin Khodosevich
Last updated by author(s): Aug 28, 2020

Reporting Summary

Nature Research wishes to improve the reproducibility of the work that we publish. This form provides structure for consistency and transparency in reporting. For further information on Nature Research policies, see our Editorial Policies and the Editorial Policy Checklist.

Statistics

For all statistical analyses, confirm that the following items are present in the figure legend, table legend, main text, or Methods section.

n/a	Confirmed	
☐	☒	The exact sample size (*n*) for each experimental group/condition, given as a discrete number and unit of measurement
☒	☐	A statement on whether measurements were taken from distinct samples or whether the same sample was measured repeatedly
☐	☒	The statistical test(s) used AND whether they are one- or two-sided<br>*Only common tests should be described solely by name; describe more complex techniques in the Methods section.*
☒	☐	A description of all covariates tested
☒	☐	A description of any assumptions or corrections, such as tests of normality and adjustment for multiple comparisons
☐	☒	A full description of the statistical parameters including central tendency (e.g. means) or other basic estimates (e.g. regression coefficient) AND variation (e.g. standard deviation) or associated estimates of uncertainty (e.g. confidence intervals)
☐	☒	For null hypothesis testing, the test statistic (e.g. *F*, *t*, *r*) with confidence intervals, effect sizes, degrees of freedom and *P* value noted<br>*Give P values as exact values whenever suitable.*
☒	☐	For Bayesian analysis, information on the choice of priors and Markov chain Monte Carlo settings
☒	☐	For hierarchical and complex designs, identification of the appropriate level for tests and full reporting of outcomes
☐	☒	Estimates of effect sizes (e.g. Cohen's *d*, Pearson's *r*), indicating how they were calculated

Our web collection on statistics for biologists contains articles on many of the points above.

Software and code

Policy information about availability of computer code

Data collection	We produced our own data and also utilized data from Allen Brain Institute available at their web-site: http://celltypes.brain-map.org/rnaseq; the description of the downloaded data is provide in the Methods part
Data analysis	We used Conos, Seurat v3 and pagoda v2 to analyze the data, the references and most important code lines are provided in the Methods part

For manuscripts utilizing custom algorithms or software that are central to the research but not yet described in published literature, software must be made available to editors and reviewers. We strongly encourage code deposition in a community repository (e.g. GitHub). See the Nature Research guidelines for submitting code & software for further information.

Data

Policy information about availability of data

All manuscripts must include a data availability statement. This statement should provide the following information, where applicable:

- Accession codes, unique identifiers, or web links for publicly available datasets
- A list of figures that have associated raw data
- A description of any restrictions on data availability

publicly available datasets were uploaded from http://celltypes.brain-map.org/rnaseq
our own datasets are available with restrictions due to EU and national legislation, thus the data are available upon request to Data availability committee

Field-specific reporting

Please select the one below that is the best fit for your research. If you are not sure, read the appropriate sections before making your selection.

☒ Life sciences ☐ Behavioural & social sciences ☐ Ecological, evolutionary & environmental sciences

For a reference copy of the document with all sections, see nature.com/documents/nr-reporting-summary-flat.pdf

Life sciences study design

All studies must disclose on these points even when the disclosure is negative.

Sample size	The analysed type of tissue is very rare and the sample size in the present manuscript (9 individual TLE samples and 10 Control samples) was determined largely by the availability of human brain samples which are either derived from surgical resection of brain tissue with particular pathology report or from post-mortem brain with low post-mortem interval (range: 1- 6h). The sample size is determined sufficient as it provides equal representation of both sexes for TLE and control samples as well as provides a good representation for different ages of TLE patients as well as epilepsy debut. Therefore, the sample size was determined sufficient to elucidate disease specific characteristics while taking into account individual variation of patients.
Data exclusions	During cell type annotation of Smart-seq2- and 10X Chromium- derived single nucleus RNA-sequencing data, nuclei expressing genes indicative of a glial cell identify were removed from further analysis as they were considered contaminants to the neuronal population of interest. Furthermore, nuclei were excluded from further analysis as doublets which showed expression of marker genes indicative of mixed cellular identities (e.g. neuronal and astrocytic).These exclusion criteria were pre-established on the basis that nuclei were isolated based on NeuN expression, thus targeting neuronal populations exclusively. Gene expression of non-neuronal cells as well as of mixed cellular identities were pre-defined to be a result of isolation impurity and therefore removed.
Replication	Due to availability of the tissue, 10X derived sequencing libraries were generated from one nuclei isolation and FACS sorting. For TLE1 which was also analyzed by Smart-seq2, single nuclei RNA-sequencing libraries were generated from nuclei isolated during six different FACS isolations of which nuclei from all Smart-seq2 plates could be amplified. Thus, the entire Smart-seq2 data set comprises of six replicates of nuclei isolation from the same brain sample using both gpSox6 and mainly rbSox6 antibodies in combination with NeuN-488.<br>For 10X libraries, each brain specimen was FACS sorted and nuclei were collected. For 10X libraries, due to the limited availability of the tissue, each brain specimen was FACS sorted and nuclei were collected once. However, in total, 19 sorts and 10X reactions were performed for TLE and control samples combined.
Randomization	We used in silico randomization such as random subsampling of fraction of cells, the description of subsampling is provided in the Methods part.
Blinding	n.a.<br>The overall goal of this study is to decipher transcriptional alterations and novel subtype-specific changes in TLE. In order to perform such analyses, the identity of each sample regarding TLE or control needed to be available.

Reporting for specific materials, systems and methods

We require information from authors about some types of materials, experimental systems and methods used in many studies. Here, indicate whether each material, system or method listed is relevant to your study. If you are not sure if a list item applies to your research, read the appropriate section before selecting a response.

Materials & experimental systems

n/a	Involved in the study	
☐	☒	Antibodies
☒	☐	Eukaryotic cell lines
☒	☐	Palaeontology and archaeology
☒	☐	Animals and other organisms
☐	☒	Human research participants
☒	☐	Clinical data
☒	☐	Dual use research of concern

Methods

n/a	Involved in the study	
☒	☐	ChIP-seq
☐	☒	Flow cytometry
☒	☐	MRI-based neuroimaging

Antibodies

Antibodies used	mNeuN-Alexa488: Cat.nr: MAB377X, Clone: A60. Lot nr.: 2736529 Vendor: Milipore. 1:2000.<br>rabbit-Sox6: Cat.nr: ab30455 Clone: polyclonal. Lot nr.: n.a. Vendor: Abcam. 1:2000.<br>Normal Rabbit IgG Isotype Cat.nr: Control: AB-105-C; polyclonal rabbit IgG. Lot nr.: ER 1517081 Vendor: R&D Systems. 1:1000.<br>Mouse IgG1 κ Isotype Control: 554121; Clone MOPC-21; Vendor: BD Pharmingen. 1:1000.<br>guinea pig-Sox6: Clone: monoclonal, amino acids 445–497 of mouse Sox6. 1:2000. gift from Prof. Dr. Michael Wegner, Institut für

	Biochemie, FAU Erlangen-Nürnberg. Reference publication: Stolt,C.C. et al. 2006, Dev. Cell
Validation	For all primary antibodies used, the initial validation method used is flow cytometry. If publications for sorting strategies on human nuclei were available, they were used in order to reproduce corresponding sorting populations. If no gating reference was available, isotype controls were used to define unspecific staining (e.g. rabSox6).<br>For the conjugated MAB377X, the unconjugated IgG1 κ Isotype Control 554121 was used in addition to reference publications to approximate background fluorescence. In general, MAB377X stained nuclei consistently yielded two clear and distinct populations of NeuN- and NeuN+ populations.<br><br>Following this, the gating strategy was validated by collection of different bulk nuclei fractions. Following RNA extraction, Smart-seq2 and Illumina library preparation was performed on 150pg input RNA for each of the populations and generated libraries were subjected to RNA-sequencing. Evaluation of expression of key marker genes demonstrated the enrichment of inhibitory neurons in the NeuN+Sox6+ fraction compared to the NeuN+ fraction, as well as enrichment of neuronal genes in the NeuN+ fraction as opposed to NeuN-. It also confirmed expression of NeuN and Sox6 in the expected nuclei fractions.<br><br>mNeuN-Alexa488 MAB377X: Reference for Validation is Lake, B. et al. 2016 Science supplementary figure 1a. PMID: 27339989<br>for additional references and data sheet see: https://www.merckmillipore.com/SE/en/product/Anti-NeuN-Antibody-clone-A60-Alexa-Fluor488-conjugated,MM_NF-MAB377X#anchor_REF<br>Additional reference for using MAB377X for flow cytometry on human nuclei : Luo, C. 2017 (Science): PMID: 28798132<br><br>guinea pig-Sox6: Reference for alidation of the antibody is Kozlenkov, A. 2015. NAR. Staining of cells was performed as described in Kozlenkov, A. 2016 and the staining was validated by the reproduction of a similar FACS profile as in Kozlenkov et al. 2016 in Fig. 1c. PMID: 26612861<br><br>rabbit-Sox6: Validation was done by including a rabbit isotype antibody (AB-105-C) to assess non-specific staining in the target sample. The rabbit isotype was used to set the gate to discriminate between Sox6+ and Sox6- populations.<br>For additional references for this antibody used in IHC plus data sheet see: https://www.abcam.com/sox6-antibody-ab30455.html<br><br>Mouse IgG1 κ Isotype Control: 554121<br>for application details provided by the manufacturer see: https://www.bdbiosciences.com/us/reagents/research/antibodies-buffers/cell-biology-reagents/isotype-controls/purified-mouse-igg1-isotype-control-mopc-21/p/554121<br>Reference provided by the manufacturer: https://www.bdbiosciences.com/us/reagents/research/antibodies-buffers/cell-biology-reagents/isotype-controls/purified-mouse-igg1-isotype-control-mopc-21/p/554121<br><br>Normal Rabbit IgG Isotype AB-105-C<br>for application details provided by the manufacturer see: https://www.rndsystems.com/products/normal-rabbit-igg-control_ab-105-c<br>Application references provided by the manufacturer: https://www.rndsystems.com/products/normal-rabbit-igg-control_ab-105-c#product-citations

Human research participants

Policy information about studies involving human research participants

Population characteristics	Population characteristics such as sex, age, disease status etc are provided in the Methods part. The selected human specimens represent different ages (18-55y) as well as varying onset of epilepsy while equally representing both sexes. In addition, the vast majority (except for TLE5) showed hippocampal sclerosis with either abnormality or potential FCD detected by MRI. The control specimens also represent both sexes equally and aim to have as low as possible PMI for best comparability to the freshly isolated TLE samples.
Recruitment	Postmortem human samples are derived from Human Tissue Brain Bank – Semmelweis University, biopsies are derived from from surgical operations performed at Departments of Neurology and Neurosurgery at Rigshospitalet
Ethics oversight	Ethical Committee in the Capital Region of Denmark (H-2-2011-104)<br>Human Tissue Brain Bank – Semmelweis University (HBTB) for brain autopsy and use of material and clinical information for research purposes based on the permission No. 6008/8/2002 and No. 32/1992/TUKEB authorized by the Committee of Science<br>and Research Ethics of the Ministry of Health, Hungary and the Regional Committee of Science and Research Ethics of Semmelweis University

Note that full information on the approval of the study protocol must also be provided in the manuscript.

Flow Cytometry

Plots

Confirm that:

- [x] The axis labels state the marker and fluorochrome used (e.g. CD4-FITC).
- [x] The axis scales are clearly visible. Include numbers along axes only for bottom left plot of group (a 'group' is an analysis of identical markers).
- [x] All plots are contour plots with outliers or pseudocolor plots.
- [x] A numerical value for number of cells or percentage (with statistics) is provided.

Methodology

Sample preparation	Sample processing for all human brain samples was identical and started from sub-dissected cortical tissue blocks (3 mm3), which were subjected to dounce-homogenization for nuclei isolation. Following isolation and filtration through a 70uM sterile filter, unspecific antibody binding was reduced by blocking the isolated nuclei using BSA. Following blocking, Sox6 and/ or NeuN antibodies were added to isolated nuclei as well as gating control antibodies (Normal Rabbit IgG Isotype and mouse IgG k Isotype). Nuclei were washed post primary antibody incubation and in case of Sox6 and rabbit Isotype control, nuclei were incubated with Alexa-647 secondary antibody. Importantly, mouse IgGk isotype was incubated with Alexa-488 as this antibody is non-fluorophore conjugated as opposed to mouse NeuN-488 conjugated antibody. Mouse IgGk isotype was therefore used as an approximation for unspecific NeuN-488 staining. Nuclei were washed and resuspended in FACS buffer and directly used for flow cytometry and isolation for either Smart-seq2 or 10X. Detailed information about sample preparation and staining conditions can be found in the methods section.<br>All tissue used is of neocortical origin and was derived either through surgical resection (TLE cases) or obtained from autopsies (Control cases). The parts of the temporal cortex that are dissected during biopsies or autopsies include Brodmann areas 20/21/22/38. The age range for TLE cases was 18 to 55 years and for controls 26 to 67 years. TLE cases comprised four male and five female brain samples whereas controls were comprised of two male and two female specimens. In order to ensure best possible comparability between biopsy and autopsy samples, the post mortem intervall (PMI) was selected as short as possible (range: 1- 6h). Non-epileptic temporal cortex from biopsies was not availble since such resections were not carried out during the time this study was performed.<br>The FACS data are shown both as Dotplots with pseudocolor as well as with additional contour plots in pseudocolor visualizing NeuN- and NeuN+ staining for 10X as well as NeuN+Sox+ double staining for Smart-seq2.
Instrument	Instruments used for data collection are BD FACS Aria I and III.<br>Serial number Aria I: P22300102<br>Serial number Aria III: P64828200088
Software	Software on the FACS Arias were used for analysis.<br>Software on Aria I: FACSDiva software version 6.1.3<br>Software on Aria III: FACSDiva software version 8.0.2
Cell population abundance	For NeuN+Sox6+ stained human nuclei, NeuN+ populations ranged from 3.4- 26.2% and NeuN+Sox6+ populations ranged from 0.2- 6.5% of the parent gate. This corresponds to a mean population size of NeuN+ (16.2+/- 8.8 SD) and NeuN+Sox6+ (3.5+/-2.1 SD). Nuclei used for the generation of the Smart-seq2 data set were obtained following this gating strategy.<br>For 10X genomics, high nuclear throughput is essential so that single cell sequencing libraries were obtained from the larger NeuN+ fraction. When staining nuclei for NeuN alone, two populations of NeuN- and NeuN+ nuclei are observed as shown in the reference publication Lake, B. et al. 2016 in Science. The fraction of NeuN+ nuclei ranged from 10.1- 63.9% for individual human brain samples. No correlation between biopsy and autopsy samples was observed regarding NeuN+ fraction size.
Gating strategy	First, FSC-A against SSC-A was used to discriminate nuclei from debris. FSC-H against FSC-W was then used to select single events. Out of all single events, all 7-AAD+ nuclei were selected. All single, 7-AAD+ nuclei were furthermore used to identify fluorescence intensity of the stained markers NeuN (and Sox6 for Smart-seq2). Rabbit isotype antibody was used to approximate the level of unspecific fluorescence signal for Sox6 staining and mIgG k was used to approximate background signal for the NeuN staining. Nuclei were either sorted into lysis buffer as single nuclei to proceed directly with Smart-seq2 (NeuN+Sox6+) or collected in bulk (NeuN+, NeuN-) to proceed immediately with the 10X workflow. Thus post-sort assessment of purity of different fractions was not assessed by flow cytometry but computationally after single nucleus RNA-sequencing. Gating strategy for NeuN sorting for 10X analyses as well as Smart-seq2 is provided in Supplementary Figure 1a and 1f, respectively.

- [x] Tick this box to confirm that a figure exemplifying the gating strategy is provided in the Supplementary Information.
